# Fighting for survival: persons with disabilities’ activism for the mediatisation of COVID-19 information

**DOI:** 10.1177/1329878X20967712

**Published:** 2021-02

**Authors:** Nhamo Anthony Mhiripiri, Ratidzo Midzi

**Affiliations:** Midlands State University, Zimbabwe

**Keywords:** ableism, access to information, activism, Deaf and Hard of Hearing, disability media studies, health communication, lawsuits, persons living with disabilities, social model of disability

## Abstract

Crises times have an uncanny way of giving salience to struggles for democracy. The new coronavirus – also known as COVID-19 – became a global public health issue that stirred other democratic concerns from persons living with disabilities who wanted access to health information for their survival. People living with various types of disabilities have special communication and information needs, some of which require specific technologies, formats and language. The pandemic got people concerned about their safety and survival. This article contextualises and critiques US, Britain and Zimbabwean activists representing persons living with disabilities’ reactions to the manner their public authorities availed COVID-19 health messages to disabled constituencies via mainstream television. It compares how suitable was televised content from US, Britain, Zimbabwean and New Zealand stations for the Deaf and Hard of Hearing, before exploring complaints and lawsuits from the disability constituency pertaining to access to COVID-19 health information.

## Introduction

In times of crises and disasters, a lack of access to communication and information platforms for people living with various types of disabilities makes them more vulnerable and prone to life-threatening situations (Kent and Ellis, 2015). This is regardless of the existence of contemporary complex and overlapping information and communication platforms, including digital ones. Disabled persons’ failure to access communication platforms and vital information during global times of crises is traceable to social, technical and affordability (financial) reasons (Kent and Ellis, 2015; Shava, 2017). An ableist culture is often blamed for giving low priority to people with disability and favouring able-bodied persons in providing services in these critical times of crises and disasters; hence, able-bodied people are implicated in making decisions that disproportionately negatively impact persons with disabilities, instead of providing best practice accessibility that benefits everyone (Kent and Ellis, 2015; Rohwerder, 2013). When communication and information channels fail to represent properly the interests and needs of persons with disabilities, a vigorous activism championing for social justice makes demands in both practice and academia (Blanck and Flynn, 2017; Goggin, 2014; Lawson and Priestley, 2017; Shava, 2017). Activism for disabled persons’ right to proper media representation and access to information and communication platforms has surged globally since the 1970s. There is an equally burgeoning global research and literature on disability studies and related aspects such as disabilities nexus with media studies, law and human rights, physical architecture and representation (Goggin, 2014; Goggin et al., 2019; Hadley and McDonald, 2019; Kent, 2019; Shava, 2017; Watermeyer et al., 2019). This article intends to ascertain how Deaf persons and those with hearing impairments accessed information communicated by public health communicators about the coronavirus (also known as the novel COVID 19) on selected mainstream television channels in the United States, the United Kingdom, New Zealand and Zimbabwe, in the first 5 months of 2020 when the pandemic dominated the global media platforms. Where there was disgruntlement about access to information from public health communicators, disability-rights activism culminated in litigation. Disabled persons, and especially the Deaf and Hard of Hearing (DHH), demanded the right to representation and access to information on COVID 19 in the United States, the United Kingdom and Zimbabwe, where there was manifestation of public activism against perceived injustices in the provision of information. The article evaluates the correlation of the type and nature of presentation of Sign Language (SL) for the DHH – or the lack of it – and the legal actions taken by activists to demand social justice in the United States, the United Kingdom, Zimbabwe and New Zealand.

This article contextualises and critiques US, Britain and Zimbabwean activists representing persons living with disabilities’ reactions to the manner their public authorities availed COVID-19-related health messages to disabled constituencies via mainstream television. We compared televised content from US, Britain, Zimbabwean and New Zealand stations to ascertain socio-political and ethical implications of their screening variations. The World Health Organization’s (WHO) public announcements on COVID-19 are also briefly studied. People living with various types of disabilities have special communication and information needs, some of which require specific technologies, formats and language. The manner in which people with disabilities, especially those with visual disabilities and impairments, the Deaf and those hard of hearing, received or did not receive public health communication on COVID-19 became a discursive issue in Zimbabwe, Britain and the United States. This is notwithstanding Zimbabwe and the rest of the countries’ disparities in levels of economic development, resources availability, institutional structures, traditions and reputations of democracy and good governance.

The COVID-19 health issue invoked discussions on the right to know, access to information and freedom of expression. International declarations and conventions as well as national constitutions are forthright in their aversion of any forms of discrimination against persons based on race, class, gender, sex, creed, nationality and so on. The same philosophy informs the principle that people with disabilities must not suffer discrimination. The COVID-19 compels societies to reassess their sincerity in respecting human rights and standards that are enshrined in various lofty agreements. The global consensus on the observation of various rights requires that there be actual respect and recognition of such rights. It is within the context of consensual rights and the existent pandemic that people living with disabilities demand that they access life-saving information timeously. Ideally, that information should be availed to these people with special needs simultaneously as it is availed to those without disabilities.

The institutional structures for providing adequate and timely space and information pertaining to the COVID-19 were found wanting during the formative stages of the spread of the disease and during its progression. Global nations adopted proactive health intervention policies of spreading mass information, and encouraging behavioural changes and adjustments, admonishing citizens on social distancing to prevent cross infection, notifying on the capacities of the existing health institutions, sharing statistics on infections and fatalities and updating on the impact to the national and international economies. People who rely on non-oral SL largely complained that they did not receive information easily and timely during the crisis – hence put at serious risk, compromising their right to safety and health. In some instances, complaints escalated to litigation against public authorities and institutions.

The COVID-19 pandemic had repercussions in the media and communication terrain for the visually disabled and impaired, the DHH in both affluent and struggling economies, as well as across countries with disparate communication structures and cultures. Some countries traditionally regarded as democratic and respectful of the rights of communication and access to information such as the United States found themselves under heavy criticism similar to what is often associated with the so-called authoritarian countries. That COVID-19 thrust itself in the public realm as a scourge blind to class, racial, gender, age and other distinctions – a leveller through and through – hence has implications for how it is mediatised. We try to establish how communication structures of the different countries responded to the virus to serve the constituencies of disabled persons.

## Theoretical framework

Since disability manifests in multiple forms and contexts, person(s) living with a specific type of disability require different information and communication technologies and formats for them to access content (Couldry et al., 2018; Ellis, 2017; Ellis and Kent, 2017). The Deaf and those living with hearing impairments thus largely rely on SL and technologies that mass mediate content into visual images or other assistive technologies for hearing. Democratisation of media and information platforms for social justice entails enabling effective media access in all its facets, inclusive of ‘distribution of media resources’ (Couldry et al, 2018: 3). There is an assumption that information is easily available and accessible to the entire world population (Fuchs, 2011). However, this supposition overlooks the realities of the critical political economy of information and media (Fuchs, 2011; Fuchs and Sandoval, 2014), and availability of resources and technologies and skills that ensure that all the people who might want to access information in real time and space can do so.

The information and communication requirements of the disabled persons’ socio-cultural group entail consideration of various theoretical lens emanating from different disciplines. There are various types of disabilities, and such disabled persons’ interactions with communication and access to information are equally complex. Hence, this study draws from different approaches from different traditions and disciplines; disability studies, inclusion and inequality research (Goggin et al., 2019; Park, 2017; Shava, 2017) disability and health communication (Kirklin, 2007a, 2007b; Lee, 2013): disability human rights and activism (Blanck and Flynn, 2017; Goggin, 2014; Lawson and Priestley, 2017; Shava, 2017) and disability media studies (Ellis et al., 2020; Ellis and Kent, 2017). There is inevitable interconnection of these approaches in spite of their apparent diverse genealogical origins. In fact, they may appear at times to conflict, such as when disability studies is juxtaposed to health communication, with the latter’s roots in medical sciences. Disability studies is an emergent discipline traceable to around the 1970s when disability activism emerged in the United Kingdom. Most research and literature of disability studies perceive disability as a socio-cultural construct. Largely using the Social Model Approach, disability theorists consider disability as a resultant idea of how dominant able-bodied (ableist) people create their mental and affective world. Disability studies therefore are preoccupied with the ideological, architectural, representational and institutional systems that negatively affect the lives of people living with physical, emotional and psychosocial disabilities (Hadley and McDonald, 2019). Applied medical sciences, which has a longer history of studying disability, has, however, perceived disability as a problem requiring a medical cure or solution. A person with any disability is thus viewed as not human enough, and quite often, this results in discrimination and marginalisation. Critical disability studies, the disability-rights movement and the related disability activism often reject the domineering and patronising assumptions of applied health sciences. A committed scholarship that straddles disability studies, arts, culture and media studies and so forth provides compelling accounts and insight(s) of the way disability is defined in dominant oppressive cultural systems, institutions and discourses (Couldry et al., 2018: 2: Ellis et al., 2020). The disability social model is consistently used to unveil the prejudices that disabled people experience in all their lives and in many contexts and situations. According to Laura Misener et al. (2019), the social model of disability was conceived and constructed in activism of multiple global disability movements, since ‘disability is a product of social marginalisation within the able-bodied or ableist culture, rather than a product of biological difference’ (p. 76). The minimisation or ignoring of the realities of disabilities and impairments within the social model approach remains debatable, because there is evidence that corrective efforts and use of assistive technologies to ameliorate the lives of the disabled are existential realities. A social model lens on disability focuses attention ‘on the social causes of inequality rather than on its biological causes’ (Lawson and Priestley, 2017: 7), but it is impossible to separate the social causes from the disadvantages emanating from the physical impairment; hence, Tom Shakespeare (2006: 3) has argued, ‘people are disabled by society and by their bodies’. The struggle for disability rights at times expresses itself in the language of ‘disability rights’ and ‘disability-power’ approaches, which both have affiliations to the social model of disability.

Strains of the social model of disability approach are traceable in what some call the ‘disability-rights’ and the ‘disability-power’ approaches (Shultz, 2000). The underlying denominator in both is that disabilities activists desire to improve the quality of lives for the disabled people, and that they attain social justice and inclusion in participating in various socio-political, economic and cultural areas. The conception of disability influences the symbolic expression of the condition, and the manifestation of its experience, existence and strategic forms. How activists name and describe disability informs their struggles. Disability becomes a discursive or rhetorical social construct with conflicting perspectives that are both fascinating and confounding (Shultz, 2000). Disability activists use either the disability-rights approach or the disability-power approach, or they combine the two. The ‘rights’ advocates seek greater access ‘to mainstream social life through removal of environmental barriers (and) greater opportunities to share in the nation’s resources as active, contributing, and employed citizens’, while the ‘power activists’ argue for the elimination of stigma around disability because they are convinced ‘disability is simply a normal variation of human diversity’ (Shultz, 2000: 259). Hence, most Deaf activists who have adopted the disability-power discourse are well known for reconceptualising what it entails to be Deaf. For them, Deaf life signifies a normal, unique and valuable culture, instead of just a disabled way of life. They contest the hegemonic status of ‘ableism’ in contradistinction to its supposed ‘inferior’ other, ‘disableism’. Shultz (2000: 263) writes,

The Deaf Power movement is the attempt by Deaf activists to establish their culture, their language, and their identity as Deaf persons as valid and worthwhile . . . (D)eafness as a handicap is much more a social problem than a physiological one and . . . the ‘signing community’ is more a linguistic minority than a disabled minority.

These perspectives are well pronounced in the social movements in the Global North, but share similarities with discursive assertions on disability in the South. Zimbabwean activists package demands for equitable media representation and access to information in virtually identical discourse (Nkomo, 2014; Shava, 2017). They combine the rights and power approaches and use them conveniently, apparently placing more significance on disability rights probably due to the level of development of the country where the disabled’s rights still require better recognition.

Disability activism coupled with the emergence of Internet as a mainstream technology and its proliferation especially in the Global North have spurred the recognition of disability as a normal, natural and worthwhile part of social life (Goggin, 2014). People with disabilities have enhanced their communicative capacities using new ICTs. However, the optimism that arose from the twin processes of technological advancement and a vigorous activism for persons with disabilities to access resources and spaces that enhance their lifestyles continues to be challenged by the equally dogged mainstream culture of ableism. According to Kent et al. (2019: 7) disability studies, alongside its radical version critical disability studies that aspires for social justice for disabled persons.For the social justice movement and other scholarly disciplines, the epistemological and practical objective is to bring wholesome change for the disabled persons and all other people. This scholarly and activist movement can then become ‘a historical footnote’ referred to in retrospect, which unfortunately it is not at the moment.

The prevalence of injustices against disabled persons means the political commitment of the social model of disability remains relevant. Thus, other critical disciplinary approaches can only augment the efforts of critical disability studies and activism. It can find an epistemological ally in critical health communication studies (Chasi, 2014; Kirklin, 2007a, 2007b; Lee, 2013; Tomaselli and Chasi, 2011). The literature locates the significance of assistive technologies and architectures in enabling the disabled people to live better lives. Unfortunately, the reproduction of systemic inequality denies the full realisation of the noble objective of making all disabled persons assess the necessary services, (digital) technologies and use of information and media (Ellis et al., 2020). This has resulted in the placing at the centre the fight for human rights of disabled persons. Such struggles largely draw their intellectual objective and vision from the Universal Declaration of Human Rights, the Convention on the Rights of Persons with Disabilities (CRPD) and the disability rights manifestos pronounced since the 1970s to date (Couldry et al., 2018; Ellis et al., 2019; Kent et al., 2019).

## The freedoms and human rights for persons with disabilities

Under the Universal Declaration of Human Rights, people living with disabilities are accorded full rights and freedoms just as any other people. The United Nation’s CRPD, however, directly recognises the unique peculiarities of such people. The CRPD obliges State Parties to respect the rights and freedoms of persons with disabilities and to ensure that public resources and necessary technologies are availed and used to realise the enjoyment of those basic rights. Communication in its diverse forms, languages and technologies, including braille, tactile communication, large print, accessible multimedia, signed languages and other forms of non-spoken languages, are a right for persons with specific types of disabilities. Such persons should not be discriminated through any means of distinction, exclusion or restriction. They should enjoy all their human rights and fundamental freedoms in the political, economic, social, cultural, civil or any other field, like any other persons, without the purpose or effect of impairing or nullifying the recognition, enjoyment or exercise of those rights and freedoms (see CRPD Articles 2, 9 and 21).

State parties are particularly obliged to ensure that persons with disabilities enjoy freedom of expression and opinion, and access to information in ‘formats and technologies appropriate to different kinds of disabilities in a timely manner and without additional cost’. Different nation-states promulgated legislation compliant with the universal agreements on the rights and freedoms of persons with disabilities. For instance, Zimbabwe’s constitution recognised SL as one of the country’s official 16 languages. The United States has the American with Disabilities Act designed to eliminate discrimination against people with disabilities. The United Kingdom’s Equality Act prohibits discrimination based on characteristics such as being Deaf.

## Health communication, power and ethics

While health communication appears to have its roots in the medical sciences that the social model of disability and disability studies in general often criticise and derive, there is still significant contributions that come out of health communication especially during times of public health crises such as the COVID-19 pandemic. Renata Schiavo (2007) sums up health communication as a multifaceted and multidisciplinary approach with the objectives to reach different audiences and share health-related information. The main intention is to influence, engage and support individuals, communities, health professionals, special groups, policymakers and the public ‘to champion, introduce, adopt, or sustain a behavior, practice, or policy that will ultimately improve health outcomes’ (Schiavo, 2007: 7). The ‘science’ of health communication is now widely accepted as a vital component of public health in the humanities, social sciences and natural sciences (Freimuth et al., 2000). Health communication is important in curbing emerging infectious diseases and raising awareness against global threats and bioterrorism, while it encourages preventive and patient-centred approaches to health.

Without undermining the other essential components of a viable public health system, the communication component is probably the most vital mechanism for healthcare delivery (Bernhardt, 2004). It is useful in informing, influencing, motivating and ultimately achieving acceptable health behaviour. However, information alone is not enough. First, individuals must want and be motivated to live in life-sustaining ways. Finally, people must have the resources and environment that is supportive of the expected positive behaviours (Ahmed and Bates, 2013; Martin and DiMatteo, 2014), including information and communication resources.

Most studies on health communication are preoccupied with the critical areas of efficacy of health messages, and the symbolic representation (framing) of illnesses and those afflicted (Lee, 2013; Worrell, 2013). The efficacy of health messages presupposes that health communication is targeted and purposive communication whose goal is to effect positive changes in people’s lives through health promotion and disease prevention. Hence, its goals are benevolent, righteous, virtuous and altruistic (Andreasen, 2001; Guttman, 2000, 2003: Kirklin, 2007a, 2007b; Kozlowski and O’Connor, 2003; Lee, 2011, 2013; Lee and Cheng, 2010:; Seedhouse, 2004). Celebrations of the virtues of health communications tend to overlook that the quality, policy objectives and symbolic intentions of health communication are equally contestable just as any other empirical and academic pursuit. Several health communication scholars acknowledge health communication is not value free and some health endeavours, including health communication itself, are harmful and undesirable, notwithstanding its historical associations with ‘public service’ (e.g. Guttman, 2000, 2003: Guttman and Salmon, 2004). The under- and mis-representations of certain diseases and disabilities are cases in point.

Critical health communication scholarship condemns the paternalistic imposition of ‘information’ and perspectives from the so-called health and political authorities, especially during crisis times (Kirklin, 2007b; Lee, 2013). Health communicators at any level in the message dissemination structure consciously and subconsciously frame health messages in ways predetermined by their personal beliefs, attitudes and values, which in turn influences the nature of the message. The facticity of the message is therefore intricately embroiled, for good or for bad, in the personal beliefs and values of the conveyor of the information (Kirklin, 2007b). When powerful health communicators disseminate messages, it is therefore possible that persuasion is interlinked with paternalistic presumptions of both power and the privilege to know and inform. Hence, Lee is concerned with ethics of health communication. The combination of persuasion and paternalism has a bearing on speech acts with their attendant ethical implications. Receivers of messages can also be deprived of the ability to make autonomous decisions on health issues (Lee, 2013: 197).

The representation of COVID-19 content and the institutional and structural dynamics that undergird the availability of this diseases’ information for the constituencies of the disabled people is therefore intricately influenced by the perceptions of individual communicators’ values and perceptions invariably contained in topics that outwardly appear objective, factual and true. The very idea of information as truth is problematic. Hence, the information that constituent groups such as the disabled person are demanding from the dominant information systems is not necessarily true in their very nature. The political economy is predicated on a patriarchal capitalist system and media systems are no exceptions, including health communication systems. For instance, there are numerous examples of when State leaders distort health information both through ignorance or ulterior motives (Chasi, 2014; Fukuyama, 2020; Tomaselli and Chasi, 2011).

## Research methodology

We selected complaints and lawsuits from the United States, Britain and Zimbabwe, three countries that elicit divergent views on most issues, notwithstanding respect for human rights. We evaluated the timing and nature of complaints and lawsuits submitted to state authorities by Deaf people and people with hearing impairments pertaining to the availing in real time of COVID-19 information. The constituency of persons living with disabilities is wide and diverse, thus the selection of a joint lawsuit of the Deaf and the visually impaired from Zimbabwe. Such people require different technologies, formats and language responsive to their quest to acquire messages through the mass media. The DHH are arguably more vulnerable with regard to acquisition of media content. Relying on visuals, SL may be absent, and captions too small and flirting for them to make sense if ever the screen provides them. SL is a language in its own right, although there are slight variations across countries, hence American SL (ASL), British SL (BSL), Zimbabwean SL (ZSL) and New Zealand SL (NZSL). General spoken language and action (content) requires further mediation through SL interpretation for the DHH to access it. A blind person who hears can access radio and television audio content or read braille if literate. However, someone who is both Deaf and blind requires braille or other tactile technologies of communication.

Due to the gravity of the COVID-19, public health communicators promptly took measures to inform citizens on how to behave. Government leaders assumed the role of principal health communicators of information and strategies for fighting the spread of the disease. Our media monitoring from January to May 2020 assisted to establish what supportive structures and arrangements were installed to ensure that the information from critical health information disseminators was conveyed instantly to persons living with disabilities, particularly the DHH requiring assistive technologies for receiving such information. We consistently monitored news bulletins and briefings on the following mainstream television stations: CNN, BBC World News, BBC Africa and ZBC TV. These television stations are differently owned. While they carry global news stories, they have spatially determined content. BBC covers substantial news on the United Kingdom. CNN is privately owned and focuses extensively on what transpires in the United States. ZBC is a state-owned and controlled station that prioritises Zimbabwe. We also traced the New Zealand Herald and New Zealand News that posted ample COVID-19-related audiovisual content on their home country situation.

We followed the channels consistently into the period when countries went into lockdown and the different phases engineered to mitigate or eradicate the spread of the virus. Since we were under lockdown in Zimbabwe, we watched television on a daily basis and saw State leaders making special media pronouncements over COVID-19. Monitoring broadcast patterns on television enabled us to verify the protestations by activists and lobbyists that they were not receiving timely information on the virus, thus endangering their lives. We searched the Internet and social media for reactions of special interest groups to the way pandemic-related information was disseminated to them. Google searching the keywords ‘disabled persons’ activism during Covid-19’ produced the articulations of individual activists and organisations for people living with disabilities. We purposively selected materials to study, with special focus on the DHH.

Through this monitoring, we selected blogger and activist Charis Hill’s article to represent an individual protesting voice. The United States of America’s The National Council on Disability and the National Association of the Deaf (NAD) who sent letters to the White House asking why there were no SL interpreters during the coronavirus taskforce public briefings, and those who sued New York Governor Andrew Cuomo represented organised group activism. This also applies to what motivates selection of the Zimbabwe lawsuit against ZBC and government. Due to the outrageous nature of Baltimore Mayor Jack Young’s censoring of an ASL interpreter before a public gathering, we are convinced this particular case has serious legal and ethical implications. The WHO as a global custodian of health issues was also monitored both on mainstream television stations and on WHO website posts, especially when the Secretary General addressed peoples of the world. English was the primary language for all broadcast content from which it was interpreted into SL and other formats.

The spread of the COVID-19 virus definitely galvanised the disabled people’s movements’ activism especially in the area of restructuring the dominant communication system that marginalised them. A critical methodology that is optimistic about marginalised peoples’ struggles against constrictive hegemonic forces informs this study. By bringing the communication issues of the disabled persons and the DHH to the centre, the research aspires to make its modest contribution in opening new spaces for the marginalised, placing this subgroup of peoples within the larger human struggle for equity and social justice (Denzin and Lincoln, 2018). COVID-19 is only an opportune chink put to use for prising democratised communicative spaces and equitable distribution of public information resources. Ours becomes a form of conscious selection of subject matter and deliberate ‘writing framed around acts of activism and resistance’ (Denzin and Lincoln, 2018: 29).

## Framing SL interpretation on television and online posts

This section analyses the presence or absence of SL interpretation for the DHH on selected mainstream television and web content focusing on official broadcasts or briefings from Zimbabwe, New Zealand, Britain, the United States and WHO. The content in SL is essentially an ‘interpretation’ and not ‘translation’. Taking a cue from official lawsuit documents from DHH activists, we assume translation connotes a literal transferring of information, while interpretation aspires to convey the overall message without being constrained by exactitudes of symbolic similarity. We first establish whether in any broadcast of crucial COVID-19 information coming from a public official or State leader, there was an SL interpreter in frame. Situating an interpreter in frame has technical variations with implications on timeliness and instantaneity. The size of the image of the SL interpreter in proportion to the size and positioning of the main presenter underlines significance of placement on screen. The size of the interpreter’s image has implications for visibility and ease of readability of SL.

The first scenario is when the interpreter appears alongside the health communicator and one camera contains the two undifferentiated in one frame. The two will appear next to each other sharing the same frame in equal measure, and the interpreter signs soon after the spoken words. Inclusion of the SL interpreter on an equal basis with a health communicator might appear distractive to those who rely on audiovisual communication, but it respects the DHH viewers, allowing them better visibility and readability of subject in frame. Such framing discursively avoids reducing the DHH to a subordinate status, nor to an ‘afterthought’.

The second scenario is when an interpreter is present at the scene as in the first case, but positioned slightly differently so that there is need for a separate camera to record his or her interpretation. What the second camera records is then relayed so that it is synchronised as an inset on the frame in which the communicator appears. This second scenario then makes the interpreter appear in final frame, relatively behind by some milliseconds in their interpretation as compared to the first scenario. Again, the inset is smaller within the main frame – a frame-within-a-frame. The second scenario is relatively respectful since it is trying to present information nearly simultaneously after the presentation by the health communicator, though the size of the inset is proportionally smaller in relation to the main frame. This second scenario is characterised by the resemblance of the in situ background of the frames used; it can be a wall with the same material or colour and designs.

A third scenario is when the interpretation is quite delayed and an interpreter may use a different location. The interpretation is then infused as a frame-within-a-frame just as in the second scenario. The last scenario has virtually no interpreter present in frame. The absence may be because there was never any interpreter in situ as the health communicator presented, or it is that the interpreter was present but was deliberately edited off from the frame that was transmitted to the public. In all scenarios, manipulation of technologies determines what audiences receive and whether they can create meaning or not.

### New Zealand

The first scenario is the format that New Zealand television stations – NZ News and New Zealand Herald – used consistently when their Prime Minister, Jacinda Arden, delivered statements. Of Arden’s eight briefings that we observed, all had a NZSL interpreter by her side. The interpreter was ideally positioned in frame to serve the information needs of the DHH constituency who are his or her primary audience. The PM and NZSL interpreter shared the frame in equal measure. On 4 April, the PM took time to thank and acknowledge NZSL interpreters who have been with her all the time that she addressed the nation. This is remarkable recognition of the DHH in the wake of COVID-19.

### Zimbabwe

In Zimbabwe, we observed seven briefings by the state President Emerson Mnangagwa, and one by Dr Egnas Mahomva, the Permanent Secretary for Health and Child Care. All these addresses were not made at the broadcasters’ studios. However, they were transmitted live to receivers. The two broadcasters accredited by the Zimbabwe Media Commission are state-owned and controlled by ZBC and ZTN. These are authorised to record the presidential addresses, unlike online stations such as ZimEye, Studio 7, Zim 263 and Open ParlyZw that pick up the feeds from either ZBC or ZTN and use these for their own audiences. ZTN and the online stations do not have SL interpreters. All ZBC broadcasts rely on relaying of ZSL interpretations, including when there are live broadcasts. The ZBC at best uses scenarios 2 and 3 described above, and at worst they do not have interpreters in the frames. This explains the suit that was filed at the High Court by Zimbabwe’s civil society organisations for persons living with disabilities discussed below. ZBC is also prone to glitches where, for instance, an interpreter can suddenly disappear from the frame-within-the frame due to technical faults during relaying. The ZSL interpreter inset often constitutes a ninth of the total frame, and in extreme instances, it is as small as an 18th of the total screen area. This poses challenges for visibility and readability.

### Britain

British Prime Minister Boris Johnson made more than 20 briefings and there was never a BSL interpreter in frame. This explains for the class lawsuit from Britain’s DHH people. This case is discussed below. The BBC World News and BBC Africa channels that we monitored did not insert SL interpreters for the briefings.

### The United States

In the United States, major news networks such as the ABC, CBS, Fox, NBC and CNN broadcast briefings on COVID-19. In his weekly updates, President Donald Trump never conducted a live briefing with an ASL interpreter present.^1^ The major television stations never bothered to relay ASL interpretations for their audiences. White House did not take heed of activists’ calls for government to provide an ASL interpreter to appear alongside the president. CNN’s response to calls to provide an ASL interpreter was that they did not have anyone capable of providing the service. This is confounding considering the abundance of human and technical resources in the United States, not to mention the presence of a university wholly dedicated to the DHH in the country.

Due to the US Federal governance system, respective states within the United States also conducted briefings usually addressed by the local Mayor or Governor. The network broadcasters provided the interpreters on their online transmission, a controversial situation that we will discuss below when we critically analyse the court case between activists and the Mayor of New York. President Trump’s role as a health communicator actually requires a re-evaluation of authorising political leaders to convey health messages. His opinions on health remedies and his attitudes towards the use of face masks as protective gear did not inspire public confidence. The American Centre for Disease Control intervened to correct Trump’s pseudo-scientific medical pronouncements on at least two occasions. The President’s rants against journalists and his conspiracy theory on China and COVID-19 did not serve the general purpose of fighting the disease and ensuring social stability. Erratic behaviour and misinformation from central authority can cause a dangerous crisis of trust during a pandemic. Fukuyama (2020) observes that there is no direct correlation on whether democracies do well in fighting the virus than authoritarian states. Trust in the times of COVID-19 entails citizens having confidence in their health workers and all levels of health communicators. Citizens need to have confidence that government leaders know what they are doing, serving the public interest and not short-term political interests. Trump appeared to serve the latter. He could afford to do so in a seriously polarised United States where a substantial number of Republicans believed political opponents were using COVID-19 as a means to attack Trump’s power base (Rachman and Fukuyama, 2020).

### WHO

As the global central health organisation, ideally WHO should serve the health information and communication requirements of its disparate constituencies. The WHO Secretary General, Tedros Adhanom Ghebreyesus, was the chief WHO health communicator. Ironically, WHO does not have an SL interpreter for the DHH constituency. On 22 April, the WHO condemned the stigmatisation against people living with disabilities, yet WHO inadvertently overlooked the same constituency with respect to their information and communication requirements during a life-threatening crisis. The world media conglomerates that relayed the Secretary General’s briefings largely did not inset SL interpreters. A critical institution such as WHO should use an SL interpreter and strategically position that interpreter to avoid him or her getting edited out of live and relayed broadcasts. Inclusion of an SL interpreter should not be left as the discretion of the recording news organisations. The best adoptable format for the WHO is that employed by the Prime Minister of New Zealand who did not leave anything to chance in informing people with disabilities during the pandemic. WHO is using a word for word transcriber that posts captions for briefings, but this is not adequate since it remotely satisfies the requirements of SL, considering SL is a distinct language.

## Activism and lawsuits for right to access COVID-19 information

The crisis period triggered activism from both individual activists and organisations, and in some cases, formal lawsuits were filed at courts of law. The pandemic invigorated blogger and activist Charis Hill, hence he declared, ‘(W)e activists perform well under stress’! He took it upon himself to represent his constituency that he claimed was severely neglected by the public system. He charged,

We disabled activists know no one is going to include us equitably in messaging about this latest public health threat. We have no historical evidence of inclusion, so why should we expect it to start now? . . . (M)y community . . . receives no support, validation, inclusion, or preparedness specifics when it comes to disasters. (Hill, 2020)

The Chief Executive Officer of the United States’s NAD, Howard Rosenblum, writes to Stephanie Grisham, White House Director of Communications:

From the first White House press conference on this coronavirus, the NAD has received daily complaints from deaf and hard of hearing citizens across the country asking why their President is not ensuring they are getting the same access to emergency information as everyone else. We have been directing their complaints to your office, and join in their concern for the lack of information for our community. Nearly all 50 states’ Governors have had qualified ASL interpreters next to them at their coronavirus public briefings, and we ask the same for the White House. (Rosenblum, 2020)

In a blatant show of lack of political will, White House incredibly claimed it did not have personnel with skills to interpret ASL.

Contrary to NAD’s claim that all states governors had ASL interpreters next to them during briefings, there are two remarkable cases that show otherwise. A lawsuit was filed in the Southern District Court of New York by Dennis Martinez, Douglas Nguyen, James Hallenbeck, Jill Wildberger and Disability Rights New York as plaintiff against Andrew Cuomo, Governor of New York. The plaintiff demanded that in his daily briefings on the virus broadcast to a national audience through channels such as Fox, CBS, ABC and NBC, the Governor should use an ASL interpreter whose signing should be in frame. They argued ASL is the primary language of the DHH, and providing it later online was detrimental to the rights of their constituency members since Internet was not universally accessible to some of them due to economic reasons. The Governor had started posting ASL interpretation on his website as from 27 March, but the plaintiff argued people like James Hallenbeck without computers and Internet access remained excluded from vital information. They relied on alternative sources of information, which delayed their timely access to information.

## Mayoral censorship of an ASL interpreter

A bizarre case that invited protestations from activists involved the censorship imposed on an ASL interpreter by the Mayor of Baltimore. During a press briefing on 21 April, Mayor Jack Young stopped an SL interpreter from signing the words of a homeless protester. ‘You interpret for us’, Young retorted, stopping the interpretation for Mark Council who had interrupted the press briefing together with other activists. The rebuked interpreter stood still for about 3 minutes while Council demanded from Young the relocation of homeless people from city shelters where they were in danger of contracting the coronavirus. This unique case symbolises direct censorship on an SL practitioner resulting in the blocking of information for a special interest group.

## The British lawsuit

Government made daily briefings but BSL users felt discriminated due to the absence of BSL interpreters. While watching one news briefing on 9 March on the BBC News channel, Lynn Stewart-Taylor tweeted #WhereIsTheInterpreter. She sensed she was missing ‘critical information’ due to the absence in frame of the BSL. A Twitter campaign emerged spontaneously and morphed into a class lawsuit against the British government. The lawsuit alleges the failure to provide BSL interpreters breaches Britain’s Equality Act which prohibits ‘discrimination or unfair treatment on the basis of certain characteristics – such as being deaf – is against the law’ (Rose, 2020). What is striking in Lynn Stewart-Taylor’s submission is that captions in printed English are particularly difficult to read since English is not necessarily her first language. BSL is her first language. Her argument fits well with the disability-power approach, where disability is a distinct culture no less than any other.

## The Zimbabwe lawsuit

The Zimbabwe National League of the Blind, Centre for Disability and Development Trust and Deaf Zimbabwe Trust won their lawsuit at the High Court where they had sued the state broadcasters ZBC and the Minister of Information, Publicity and Broadcasting Services and other ministries for failure to provide timely critical information in formats accessible to the DHH and blind. Since the lawsuit involved plaintiffs with different types of disabilities, the ruling had various instructions on how the DHH and the blind were to be given information pertaining to COVID-19. The High Court ordered ZBC to provide subtitles and captions for all pre-recorded programmes and a ZSL interpreter for all main bulletins and live briefings and programming. The three ministries of Information, Publicity and Broadcasting Services, Health and Child Care and Public Service and Social Welfare were ordered to produce braille pamphlets and large text with information about coronavirus. The Ministries were also to ensure all written information related to the virus provided by government, including daily updates, was availed in formats accessible to blind and partially sighted persons. This content includes audio recordings distributed on WhatsApp and/or readable digital text. Since the lawsuit was against government entities, the Minister of Information, Publicity and Broadcasting Services was instructed to ensure that privately owned media similarly complied with the directives of provision of coronavirus-related information to persons with disabilities. The lawsuits in Zimbabwe, Britain and the United States were all targeted at public entities because these are obliged under the United Nation’s Convention on the Rights of People with Disabilities, other international and state law to cater for the needs of persons with disabilities using public resources.

## Analytical reflections

Three countries considered in this article are from the Global North, and economically and technologically advanced, notwithstanding internal differences of access to opportunities that exist in these countries. Arguments in the US court cases show that some US citizens fail to use and access media due to lack of financial resources to access the Internet where alternative information on COVID-19 is available. Of course, there are large groups of people who still struggle to access the media, whether abled or disabled, and the DHH from the marginalised racial, gender and class groups are likely to experience serious need as compared to their affluent compatriots. However, Zimbabwe is in the Global South and likely to have more disadvantaged and marginalised DHH persons. More disabled persons in Zimbabwe are likely to experience needy lifestyles and lack of access to information due to lack of TV availability, Internet connectivity and affordability when the technologies are available. Electronic media does not have universal coverage in Zimbabwe, especially in rural areas where the majority reside. Radio is arguably the ubiquitous media in Zimbabwe, and television is a luxury for the majority (Information and Media Panel of Inquiry, 2014). Besides the technical access problems linked to technical literacy and affordability, Zimbabwe’s disabled people woes are compounded due to cultural attitudes as they are viewed as a curse to their families and, at worst as subhuman (Shava, 2017; Tarusarira and McKenzie, 2019), are likely to impinge on the majority of Zimbabwe’s DHH’s access to information during the COVID-19 days. The DHH from extremely poor Zimbabwean families thus are worse off during pandemic situations.

The quick spreading of the COVID-19 virus sparked vigorous activism among persons living with disabilities who largely complained that they were either virtually excluded from information that was crucial for their safety and survival in the wake of the pandemic, or the information came to them not as timely as it would have been delivered to other people. This was a general trend whether in an advanced economy such as the United States where there is the general perception freedom of the media and freedom to access to information are cardinal tenets of the developed democracy, or in Zimbabwe with its myriad problems related to resource availability and distribution, and allegations of human rights abuses (Mhiripiri, 2015). Complaints came from both individuals and organised civil society organisations representing persons with disabilities. The COVID-19 reinvigorated the civil and political rights movements in an intriguing manner. ‘Systemic ableism’ and its associated attitudes and policies against disabled people remain implicated in the inequitable provision of COVID-19 information to the DHH (O’Brien, 2020). The COVID-19 pandemic is undoubtedly a public health issue with normative and political overtones. Times of crises have an uncanny way of accentuating struggles for democracy and recognition of personhood of marginalised peoples as reflected in social movements such as the United Kingdom’s #Whereistheinterpreter, activists’ blogging and lawsuits. We are certain politically committed disability studies and disability media studies research will certainly reaffirm that activism for social justice (Kent et al., 2019; Misener et al., 2019). Deaf activists have waged notable battles in courts and in the media. More challenges are appearing in social media as has been in this article and elsewhere (Ellis, 2017). Deaf activisms that manifest as litigation for access to media content are unrelenting and timeously relate to contemporary media technologies. They have scored both legal victories and setbacks. Besides managing to acquire provision of Video on Demand for people with disabilities, activism compels public administrators, lawmakers and corporations to reflect on whether persons with disabilities are now fairly accommodated in both legacy media and new media (Ellis, 2017). More legal and media demands appear in the domain of Internet accessibility and affordability, an area that already exhibits the disproportionate marginalisation of persons with disabilities’ participation in all forms of life. There is no assurance of victory for Deaf activists in their court cases. For instance, the US Deaf activists waged a campaign against Netflix’s failure to put captions on entertainment content. The media company conceded to provide 100% caption to its catalogue after Deaf activists’ successful Americans with Disability Act (ADA) complaint. Nonetheless, a US Federal court of appeals subsequently ruled Netflix was not obliged to comply with the ADA since the company ‘was not connected to any physical place’ (Ellis, 2017: 150).

When a pandemic erupts, information is vital for people to know how to protect themselves and how to access essential goods and services during quarantine and self-isolation. The different levels of government right up to the chief executive or presidency have a public obligation to provide accurate, timely and accessible messages about the pandemic. They also clarify on the availability of scientifically proven prevention methods, remedies and institutional services to mitigate the impact of the disease. Access to such information is critical for the survival of all people. It is within the COVID-19 crisis context that people with disabilities from across the world found yet another chance to re-evaluate their status in relation to their communities and governments.

However, the discourses so far used by the activists barely reveal the need for the activists to participate in a genuine public sphere in which they exchange ideas with peer citizens in the wide society so that they contribute to how best COVID-19 should be tackled. If that quest is there, it is implicit and extremely understated. This is in spite of the fact that the disability-power sector of the disability movement insists on the articulation of their culture. Of course, persons living with disabilities have something to contribute towards the fight against COVID-19, both to save themselves as a special interest group and to protect the universal human society. Blogger and disability activist Charis Hill (2020) points out that he ‘wrote suggested language about public responsibility to prevent Covid-19 spread (“his” mayor’s office adopted the language!)’. People living with disabilities can go beyond asking for information and provide information through their preferred formats and media.

The disability-rights approach was explicitly pronounced in the demands for equal and timely access of information in formats accessible to persons living with disabilities. However, the disability-power approach remained present but in the background especially in the case against Governor Cuomo where it was apparent part of the argumentation for the plaintiffs drew from their specific lived experiences and disability culture. It is also important that the freedom of expression of persons living with disabilities and especially the freedom of their SL interpreters to conduct their work freely as conduits for the DHH must be jealously protected. Mayor Jack Young’s censoring of the SL interpreter in Baltimore reveals the potential hazards to freedom of expression in the area of mediating for persons with disabilities and must be condemned with the contempt it deserves. There is no correlation on how so-called democracies and authoritarian countries respond to the coronavirus or the respect of the information requirements of the persons living with disabilities. Lawsuits that are nearly similar in content and intent submitted in both the United States and Zimbabwe will eventually ascertain the political will of the respective governments to comply with the court rulings. Conceding that disability constitutes distinct cultural realities which may come with distinct languages thrusts disability issues into the critical terrain with disparities and inequalities that require eradication or redress (Len-Rios, 2009). Not well studied in this article is whether disability constituencies are a viable media market that can induce media producers to provide informational and media content in the supply and demand chain. However, the study is clear information and media serve a public service mandate regardless of the media business ownership and control model. Persons living with disabilities, especially the DHH, demand information on public health issues in a crisis time as a matter of life or death. Ethical considerations make such information a public service necessity rather than a class or commercial imperative.

## Conclusion

The activism for the DHH in quest of access to COVID-19 information is indeed a life or death matter considering the malevolence of the pandemic. Information and communication remain vital weapons in the fight against the disease especially at a time when there is no known vaccine. The lives of disabled people should not be prioritised less due to a selfish ableist culture. According to Kent et al. (2019: 7) disability studies, alongside its radical version critical disability studies that aspires for transformation for social justice for disabled persons, and which smoothly fuses with social justice movement and other scholarly disciplines, the epistemological and practical objective is to bring wholesome change for the disabled persons and all other people. This scholarly and activist movement can then become ‘a historical footnote’ referred to in retrospect, which unfortunately it is not the case at the moment. Generally, inroads have been made in expanding the communication spaces and access to information opportunities for persons living with disabilities. However, the optimism that this development cascades is still underpinned with the sour realisation that the struggle has not been won altogether, whether in the Global North or South. There are attitudinal and structural conditions that still militate against the complete realisation of an equitable and just society for the disabled people and the DHH in particular. Indeed, it is still time yet for struggles for social justice for the disabled to be seen in hindsight as a historical footnote.

## Notes

1. President Trump and the White House officials were sued later, although this article confined itself to cases that happened by end of May 2020 (see O’Brien, 2020).
